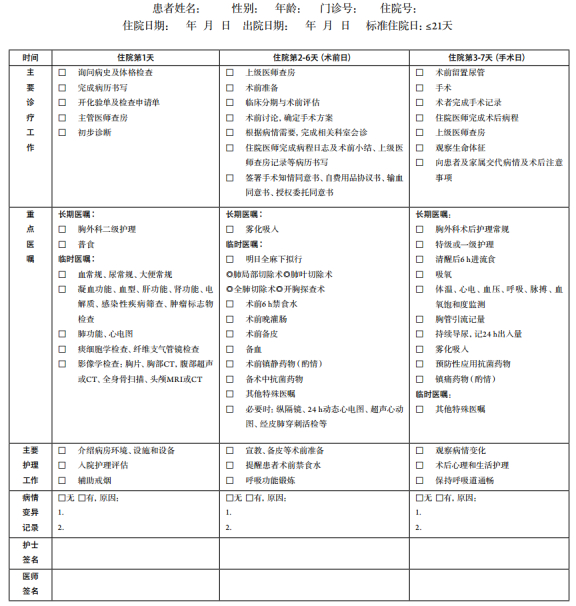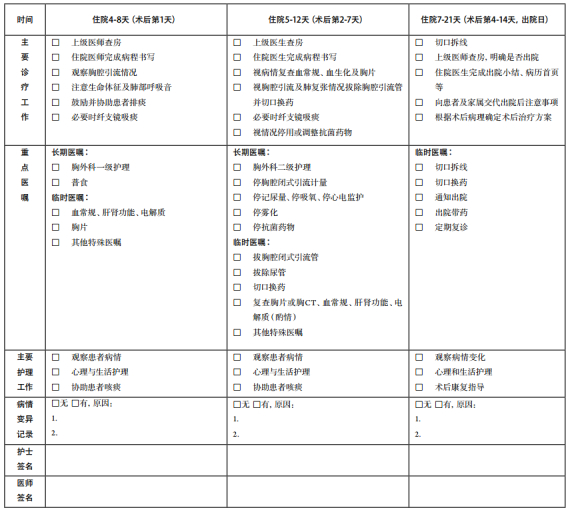# 原发性肺癌外科手术临床路径（2013年版）

**DOI:** 10.3779/j.issn.1009-3419.2013.09.01

**Published:** 2013-09-20

**Authors:** 卫生部临床路径专家审核委员会胸外科专家组, 修益 支, 健行 何, 辉 李, 逊 张, 格宁 姜, 珩 赵, 伦旭 刘, 德若 刘, 单青 李, 简 李, 清华 周, 群 王, 如文 王, 剑华 傅, 林 许, 临友 张, 乃康 周, 绍发 许, 东红 陈

**Affiliations:** 1 100053 北京，首都医科大学宣武医院 Beijing Xuanwu Hospital, Capital Medical University, Beijing 100053, China; 2 510120 广州，广州医学院第一附属医院 the First Afliated Hospital of Guangzhou Medical College, Guangzhou 510120, China; 3 100020 北京，北京朝阳医院 Beijing Chaoyang Hospital, Beijing 100020, China; 4 361000 天津，天津市胸科医院 Tianjin Chest Hospital, Tianjin 361000, China; 5 200433 上海，上海市肺科医院 Shanghai Pulmonary Hospital, Shanghai 200433, China; 6 200030 上海，上海市胸科医院 Shanghai Chest Hospital, Shanghai 200030, China; 7 610041 成都，四川大学华西医院 West China Hospital of Sichuan University, Chengdu 610041, China; 8 100029 北京，卫生部中日友好医院 Sino-Japanese Friendship Hospital, Ministry of Health, Beijing 100029, China; 9 100730 北京，北京协和医院 Peking Union Medical College Hospital, Beijing 100730, China; 10 100034 北京，北京大学第一医院 the First Hospital Afiated toPeking University, Beijing 100034, China; 11 300052 天津，天津医科大学总医院 Tianjin Medical University General Hospital, Tianjin 300052, China; 12 200032 上海，复旦大学中山医院 Zhongshan Hospital, Fudan University, Shanghai 200032, China; 13 400042 重庆，第三军医大学大坪医院 Daping Hospital, Tird Military Medical University, Chongqing 400042, China; 14 510060 广州，中山大学防治肿瘤中心 Cancer Prevention Center, Sun Yat-Sen University, Guangzhou 510060, China; 15 210009 南京，江苏省肿瘤医院 Jiangsu Cancer Hospital, Nanjing 210009, China; 16 150086 哈尔滨，哈尔滨医科大学第二医院 the Second Hospital of Harbin Medical University, Harbin 150086, China; 17 100091 北京，解放军总参谋部总医院 General Hospital of People's Liberation Army General Staff, Beijing 100091, China; 18 101149 北京，北京胸科医院 Beijing Chest Hospital, Beijing 101149, China

## 1 原发性肺癌外科手术临床路径标准住院流程

### 1.1 适用对象

#### 1.1.1 第一诊断为原发性肺癌（ICD-10: C34/D02.2）。

#### 1.1.2 临床分期（UICC 2009）为Ⅰ期、Ⅱ期、和可完全性切除的Ⅲa期非小细胞肺癌。

#### 1.1.3 临床分期（UICC 2009）为T1-2N0M0的小细胞肺癌。

#### 1.1.4 行肺局部切除/肺叶切除/全肺切除/开胸探查术（ICD-9-CM-3:32.29/32.3-32.5）。

### 1.2 诊断依据

根据卫生部《原发性肺癌诊疗规范（2011年版）》，卫生部《原发性肺癌诊断标准》（2010年版）。

#### 1.2.1 高危因素：吸烟指数 > 400年支，年龄 > 45岁，肺癌家族史等。

#### 1.2.2 临床症状：早期可无明显症状。常见的症状有：刺激性咳嗽、血痰或咯血、胸痛、气促、发热等。

#### 1.2.3 辅助检查：胸部影像学检查、血肿瘤标记物、痰细胞学检查、纤维支气管镜等。

#### 1.2.4 细胞、组织学等病理学诊断阳性为确诊标准。

### 1.3 治疗方案的选择

按照卫生部《原发性肺癌诊疗规范（2011年版）》：

#### 1.3.1 肺部分切除术（包括肺楔形切除和肺段切除）。

#### 1.3.2 肺叶切除术（包括复合肺叶切除和支气管、肺动脉袖式切除成型）。

#### 1.3.3 全肺切除术。

#### 1.3.4 上述术式应行系统性淋巴结清扫或采样。

全面的治疗计划和必要的影像学检查（临床分期检查）均应当在非急诊手术治疗前完成。充分评估决定手术切除的可能性并制订手术方案。

手术的原则是尽可能做到肿瘤和区域淋巴结的完全性切除；同时尽量保留有功能的健康肺组织。视频辅助胸腔镜手术（video assisted thoracoscopic surgery, VATS）主要适用于Ⅰ期-Ⅱ期肺癌患者。

### 1.4 标准住院日为≤21 d。

### 1.5 进入路径标准。

#### 1.5.1 第一诊断符合ICD-10：C34/D02.2肺癌疾病编码。

#### 1.5.2 心、肺、肝、肾等器官功能可以耐受全麻开胸手术。

#### 1.5.3 当患者合并其他疾病，但住院期间不需要特殊处理也不影响第一诊断的临床路径流程实施时，可以进入路径。

### 1.6 术前准备≤6 d。

#### 1.6.1 必需的检查项目：

##### 1.6.1.1 血常规、尿常规、大便常规；

##### 1.6.1.2 凝血功能、血型、肝功能、肾功能、电解质、感染性疾病筛查（乙肝、丙肝、艾滋病、梅毒等）；

##### 1.6.1.3 肺功能、心电图、动脉血气分析；

##### 1.6.1.4 痰细胞学检查、纤维支气管镜检查；

##### 1.6.1.5 影像学检查：X线胸片、胸部CT（平扫+增强扫描）、腹部超声或腹部CT、全身骨扫描、头颅MRI或增强CT。

#### 1.6.2 根据患者病情，可选择以下项目：

##### 1.6.2.1 纵隔镜或EBUS；

##### 1.6.2.2 经皮肺穿刺活检；

##### 1.6.2.3 超声心动图，24 h动态心电图；

##### 1.6.2.4 肿瘤标志物；

##### 1.6.2.5 心脑血管疾病相关检查。

#### 1.6.3 术前风险评估。

### 1.7 预防性抗菌药物选择与使用时机。

抗菌药物使用应按照《抗菌药物临床应用指导原则》（卫医发〔2004〕285号）执行。术前30 min预防性使用抗菌药物。

### 1.8 手术日为入院≤7 d。

#### 1.8.1 麻醉方式：气管插管静脉复合全麻。

#### 1.8.2 手术耗材：闭合器、切割缝合器、血管夹、止血材料等。

#### 1.8.3 术中用药：抗菌药物。

#### 1.8.4 输血：视术中出血情况而定。

#### 1.8.5 病理：冰冻切片。

### 1.9 术后住院恢复≤14 d。

#### 1.9.1 必须复查的项目：

##### 1.9.1.1 血常规、肝功能、肾功能、电解质；

##### 1.9.1.2 胸片（术后第1天和拔胸腔闭式引流管之前各1次），必要时可行胸部CT；

##### 1.9.1.3 病理检查参照卫生部《原发性肺癌诊疗规范（2011年版）》。

#### 1.9.2 术后预防性使用抗菌药物：按照《抗菌药物临床应用指导原则》（卫医发〔2004〕285号）执行。

#### 1.9.3 视病情可延长抗菌药物用药时间及更换药物种类。

### 1.10 出院标准。

#### 1.10.1 切口愈合良好，或门诊可处理的愈合不良切口。

#### 1.10.2 生命体征平稳。

### 1.11 变异及原因分析。

#### 1.11.1 各种原因导致未能在规定时间内完成术前准备；药物应用超出路径规定范围等。

#### 1.11.2 术后出现肺部感染、呼吸功能衰竭、心脏功能衰竭、支气管胸膜瘘等并发症，需要延长治疗时间或费用超出参考费用标准。

#### 1.11.3 高级职称医师认可的变异原因。

#### 1.11.4 患者以及其他方面的原因。

### 1.12 参考费用标准：3万元-5万元（VATS手术4万元-6万元）。

## 2 支气管肺癌临床路径表单

适用对象：第一诊断为支气管肺癌（ICD-10：C34; D02.2）行肺局部切除/肺叶切除/全肺切除+系统性淋巴结清扫、开胸探查术（ICD-9-CM-3:32.29/32.3-32.5）。